# Serial Immunoprecipitation of 3xFLAG/V5-tagged Yeast Proteins to Identify Specific Interactions with Chaperone Proteins

**DOI:** 10.21769/BioProtoc.2348

**Published:** 2017-06-20

**Authors:** Xu Zheng, David Pincus

**Affiliations:** Whitehead Institute for Biomedical Research, Cambridge, USA

**Keywords:** Immunoprecipitation, Yeast, FLAG tag, V5 tag, Protein complexes

## Abstract

This method was generated to isolate high affinity protein complexes from yeast lysate by performing serial affinity purification of doubly tagged 3×FLAG/V5 proteins. First, the bait protein of interest is immunoprecipitated by anti-FLAG-conjugated magnetic beads and gently eluted by 3×FLAG antigen peptide. Next, the bait protein is recaptured from the first eluate by anti-V5-conjugated magnetic beads and eluted with ionic detergent. In this manner, the majority of abundant, nonspecific proteins remain either bound to the first beads or in the first eluate, allowing pure, high affinity complexes to be obtained. This approach can be used to show specific interactions with notoriously ‘sticky’ chaperone proteins.

## Background

**Figure 1:**
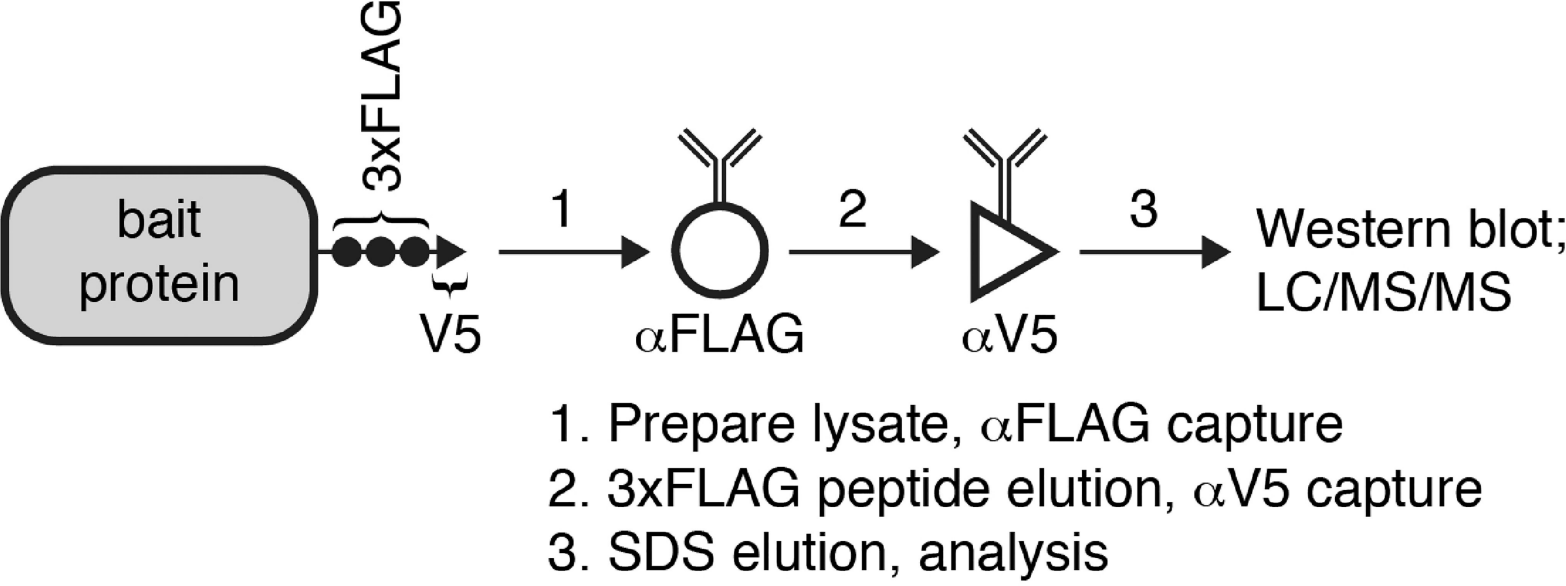
Schematic overview of the protocol A 3×FLAG/V5 dual-tagged bait protein is serially purified with anti-FLAG beads followed by anti-V5 beads.

Immunoprecipitation followed by mass spectrometry (IP/MS) is an unbiased method to identify protein-protein interactions with a specific bait protein of interest. While this approach has been fruitfully applied to identify protein interaction networks, it is plagued by false positives–proteins that appear to interact but are actually non-specifically bound to the beads or antibodies used in the affinity purification. In particular, highly abundant proteins such as ribosomal proteins, metabolic enzymes and chaperone proteins are common contaminants. However, sometimes these common contaminants may be bona fide interaction partners, yet it is challenging to demonstrate specificity. To overcome this obstacle, we developed a serial affinity purification approach to isolate specific, high affinity complexes between bait proteins of interest tagged with two affinity epitopes–the 3×FLAG and V5 tags (Figure 1). We have generated a plasmid containing the 3×FLAG-V5 epitopes and a *HIS3* selectable marker that can be amplified and used to C-terminally tag any yeast protein of interest in a single yeast transformation (Zheng *et al*., 2016). We originally applied our method to demonstrate a specific interaction between the heat shock transcription factor (Hsf1) and the major Hsp70 chaperone proteins present in the yeast cytosol, Ssa1/2. However, this approach can be generally applied to identify high affinity complexes involving a protein of interest. While this technique removes the bulk of false positive interactions, a major caveat is that low affinity and transient interactions are likely to be lost.

## Materials and Reagents

1. Pipette tips
2. Glass culture tubes (20 × 150 mm) (Sigma-Aldrich, catalog number: C1048)
3. 50 ml Falcon tubes
4. 1.5 ml microcentrifuge tubes
5. *Saccharomyces cerevisiae* (W303 background) with 3×FLAG-V5 tagged bait protein of interest (Hsf1-3×FLAG/V5 in this protocol–Pincus lab strain DPY118: *MATa ADE2 leu2-3,112 can1-100 ura3-1 his3-11,15 hsf1*Δ*::KAN HSF1-3×FLAG/V5::TRP1*)
6. 3×FLAG peptide (Sigma-Aldrich, catalog number: F4799)
7. Liquid nitrogen
8. Dry ice pellets
9. Anti-FLAG magnetic beads (Sigma-Aldrich, catalog number: M8823)
10. Anti-V5 magnetic beads (MBL International, catalog number: M167-11)
11. 2% SDS
12. Yeast extract (RPI, catalog number: Y20020)
13. Peptone (RPI, catalog number: P20240)
14. Glucose (Sigma-Aldrich, catalog number: G8270)
15. Dextrose (Sigma-Aldrich, catalog number: D9434)
16. HEPES (Sigma-Aldrich, catalog number: H3375)
17. Sodium chloride (NaCl) (Sigma-Aldrich, catalog number: S7653)
18. Triton X-100 (Sigma-Aldrich, catalog number: X100)
19. Deoxycholate (Sigma-Aldrich, catalog number: D6750)
20. Ethylenediaminetetraacetate acid (EDTA) (Sigma-Aldrich, catalog number: EDS)
21. cOmplete Mini EDTA-free protease inhibitor (Roche Molecular Systems, catalog number: 11836170001)
22. YPD media (see Recipes)
23. IP lysis buffer (see Recipes)

## Equipment

1. Pipettes
2. Incubator shaker (*e.g.*, Eppendorf, New Brunswick™, model: Innova^®^ 44, catalog number: M1282-0004)
3. 500 ml flask
4. Spectrophotometer (*e.g.*, Thermo Fisher Scientific, Thermo Scientific™, model: Orion™ AquaMate 7000, catalog number: AQ7000)
5. Centrifuge for 50 ml Falcon tubes (*e.g.*, Thermo Fisher Scientific, Thermo Scientific™, model: Sorvall™ Legend™ XT, catalog number: 75004505)
6. Cryogenic tissue grinder (Bio Spec Products, catalog number: CTG111)
7. 200 ml glass beaker
8. Intelli-mixer rotating mixer (Labscoop, catalog number: EL-RM2L)
9. Magnetic separation rack (New England Biolabs, catalog number: S1506S)
10. Thermomixer (Eppendorf, model: ThermoMixer^®^ C, catalog number: 5382000023)
11. Vortex mixer (VWR, catalog number: 97043-562)

## Procedure

1. Inoculate the yeast strain harboring Hsf1-FLAG-V5 in a culture tube containing 3 ml YPD liquid media (see Recipes) and grow over night at 30 °C in a shaker set at 200 rpm.
2. Dilute 2 ml overnight grown culture into the 500 ml flask containing 100 ml YPD media, and grow to OD_600_ = 0.5–0.8 OD at 30 °C in a shaker set at 200 rpm (~4 h).
3. Pour the growth media into two 50 ml Falcon tubes, and centrifuge at 4,000 × *g* for 4 min. Discard the media and submerge the Falcon tubes containing the yeast pellets in liquid nitrogen. The pellets can be stored at −80 °C for up to 3 months.
4. Add dry ice pellets into a cryogenic tissue grinder pre-chilled at 4 °C (a simple coffee grinder will work as well) and grind it into powder that covers the blades. The dry ice allows the cells to be lysed while remaining frozen. Dry ice should be handled with care and never confined in an airtight compartment.
5. Add the yeast pellets to the crushed dry ice and grind the yeast pellet with the dry ice for 30 sec.
6. Repeat the grinding 5 more times for a total of 6 grindings. Add more dry ice pellets as needed to keep the blades fully covered with dry ice.
7. Transfer the pulverized cells/dry ice powder into beaker, and sublimate/evaporate dry ice at room temperature.
8. Add 1 ml lysis buffer and incubate the lysate at 4 °C for 5 min with intermittent swirling to resuspend.
9. Transfer the lysate into a 1.5 ml centrifuge tube, and centrifuge at 20,000 × *g* for 10 min. Reserve the supernatant (cleared lysate). Take a 10 µl sample of the cleared lysate for analysis by Western blot (input sample).
10. Add 25 µl anti-FLAG magnetic beads to a 1.5 ml tube. Place the tube on a magnetic separation rack and remove the buffer. Wash with 200 µl lysis buffer (see Recipes) on the magnetic rack and discard the wash.
11. Pipet the cleared lysate into the 1.5 ml tube containing the anti-FLAG beads and incubate at 4 °C for 2 h on an inversion rotating mixer.
12. Place the tube on the magnetic separation rack. When the solution clears (~2 min), take a 10 µl sample for analysis by Western blot (unbound sample).
13. Discard the unbound fraction and wash the beads 3 times with 500 µl lysis buffer. Gently vortex the beads with each wash step and incubate on ice for 5 min. Return to the magnetic separation rack and discard washes.
14. Elute the Hsf1-FLAG-V5 complex with 500 µl lysis buffer containing 100 µg/ml 3×FLAG peptide. Incubate on ice for 30 min. Repeat for a total of 2 elutions, and pool the eluate fractions.
15. Add 25 µl anti-V5 magnetic beads to a 1.5 ml tube. Place the tube on a magnetic separation rack and remove the buffer. Wash with 200 µl lysis buffer on the magnetic rack and discard the wash.
16. Pipet the pooled 3×FLAG eluate into the tube with the V5 beads and incubate at 4 °C for 2 h on an inversion rotating mixer.
17. Place the tube on the magnetic separation rack. Discard the unbound fraction and wash the beads 3 times with 500 µl lysis buffer. Vortex the beads at half-maximal setting for 5 sec with each wash step and incubate on ice for 5 min. Return to the magnetic separation rack and discard washes.
18. Add 100 µl lysis buffer + 2% SDS. Incubate for 15 min at 95 °C. Place tube on the magnetic separation rack and reserve solution.
19. Use 10 µl for analysis by Western blot (final eluate: this is 10× concentrated compared to input and unbound fraction).
20. Submit remaining 90 µl samples to a mass spectrometry core facility (*e.g.*, Whitehead Institute Proteomics Facility) for analysis as described (Zheng *et al*., 2016). Mass spectrometry is extremely sensitive to contamination by proteases, so wearing gloves, working quickly and keeping samples on ice prior to analysis is of paramount importance.

## Data analysis

**Figure 2:**
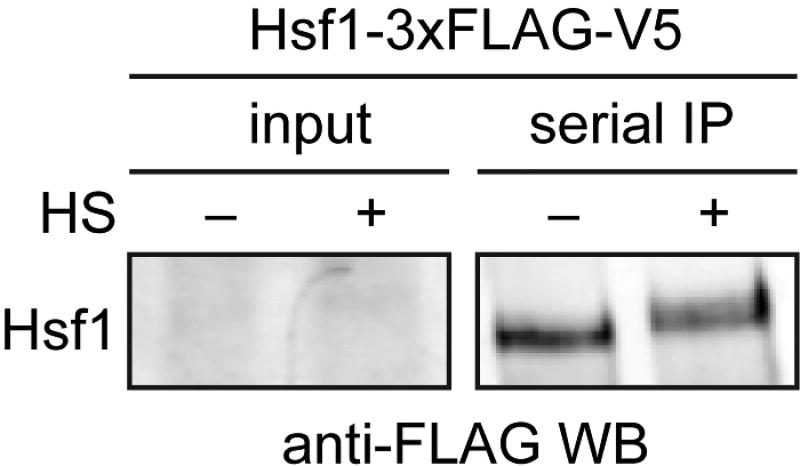
Western blot of immuno-precipitated Hsf1-3×FLAG/V5 3×FLAG/V5-tagged Hsf1 was serially purified with anti-FLAG and anti-V5 beads from cells under control (−) and heat shock condtions (+). Hsf1 is low abundance and cannot be easily detected in the input, but is enriched following immuno-precipitation. Hsf1 migrates slower in the gel under heat shock conditions due to phosphorylation (HS = heat shock).

Mass spectrometry data should be analyzed by first ensuring that you observed your bait protein with reasonable coverage (> 25%). Putative interacting proteins should be validated by repeated experiments and alternative detection methods, such as Western blotting (Figure 2). For examples of peptide counts of a bait protein, binding partners and nonspecific contaminants, see Figure 1–source data 1 in Zheng *et al*., 2016.

## Notes

Despite the stringency of the serial affinity protocol, background contaminants will still be observed in the mass spectrometry data. In general, the repertoire of highly abundant ribosomal proteins and metabolic enzymes that are nonspecific contaminants is not very reproducible, though a subset is always present. Thus, with enough replicates, most of these contaminants can be discarded.

## Recipes

1. YPD media
  1% yeast extract
  2% peptone
  2% glucose
  Autoclave before adding glucose or filter sterilize
2. IP lysis buffer
  50 mM HEPES pH 8.0
  150 mM NaCl
  1% Triton X-100
  0.1% deoxycholate
  5 mM EDTA
  1× cOmplete Mini EDTA-free protease inhibitor
